# Positional biases in social behaviors: Humans vs. saiga antelopes

**DOI:** 10.3389/fnbeh.2022.1103584

**Published:** 2023-01-09

**Authors:** Andrey Giljov, Karina Karenina

**Affiliations:** Department of Vertebrate Zoology, Faculty of Biology, Saint Petersburg State University, St. Petersburg, Russia

**Keywords:** behavioral lateralization, social behavior, ungulates, artiodactyls, human behavior, side bias, visual lateralization, cradling

## Introduction

The current evidence indicates that the lateralized processing of social information is an integral part of the perception of the conspecifics (e.g., Deng and Rogers, 2002; Kendrick, 2006; Baraud et al., 2009) which may significantly impact the cost-benefit ratio of the particular individual involved in the social interaction (Bisazza and Dadda, 2005; Jennings, 2012; Krakauer et al., 2016; Camerlink et al., 2018). One-sided biases in the behavior of individuals involved in social interaction are probably the most well-studied naturally-occurring manifestation of lateralized social processing. Lateral preferences in spatial positioning, relative to a visible stimulus, usually arise from the asymmetrical use of the lateral visual fields of the left and right eye, which, in turn, is underpinned by hemispheric lateralization (Rogers, 2017). In the social context, this means that the preferential choice of a particular lateral position relative to a conspecific reflects an individual's preference to keep the social partner in its left/right hemispace, implying the advantage of the contralateral hemisphere in social processing.

Lateralization of social cognition has been studied on a wide range of vertebrates. Across mammals, lateralized social behaviors were investigated not only in humans and other primates but also in a variety of non-primate species (Rosa Salva et al., 2012; Goursot et al., 2021). Nevertheless, social lateralizations in primates and, in particular, humans have long been analyzed somewhat in the isolation from the other mammalian taxa (e.g., Basile et al., 2009; Lindell, 2013). This could be explained by more sophisticated methods of primate research (Brancucci et al., 2009) which are barely comparable to those in often observational non-primate mammal studies. Nowadays the relevance of more naturalistic research in the field of behavioral lateralization is becoming more clear (Manns, 2021). A less intrusive, observational approach may increase comparability between human and non-human studies. As the number of such studies is growing, it may be a good time to start looking at possible similarities or differences between the taxa more closely than before. Among non-primate mammals, ungulates, with a diverse range of social interactions studied, may serve as a particularly useful model for comparison with primates. Here we briefly summarize and juxtapose some of the key findings about the lateral biases in the positioning of interacting individuals in humans and ungulates. We also discuss the importance of the wider use of non-primate models in general, and ungulates in particular, in the studies of social lateralization.

### Positional biases in human social behavior

Positional biases in human social behavior have long been known from the cradling bias, a preference to position an infant on the left rather than the right side of the body (Salk, 1960). This intriguingly universal bias has been confirmed using a variety of methods from observations in maternity hospitals to imagined cradling tasks and is evident not only in mothers but in nulliparous female adults, female children (reviewed in Harris, 2010), and even adult males (Herdien et al., 2021). Left-side bias in infant cradling has also been found in captive gorillas and chimpanzees (Manning et al., 1994) but not in other primates studied (Hopkins, 2004).

Not only the behavior directed to a child is lateralized, but children themselves show positional biases toward the adults. Unobtrusive observations of children spontaneously choosing navigational routes around adults showed that a rightward navigational path favoring the left visual field use was preferred (Forrester et al., 2014). A similar tendency was evident in the case of age-mates, i.e., children preferred to keep peers to the left side when moving around them.

The interactions between adult humans also became the object of laterality research. Observations in airports revealed that embraces in human adults demonstrate a rightward bias as indicated by the arm that was leading the embrace (Turnbull et al., 1995). Observations on lip-kissing couples in public places have repeatedly revealed rightward head-turning preference (Güntürkün, 2003; Barrett et al., 2006; Karim et al., 2017). In contrast to romantic kissing, an analysis of images on the Internet showed a left-turn bias for parental kissing (Sedgewick and Elias, 2016). The study of cheek kisses used as a formal greeting showed that the majority of individuals within one city show a consistent bias, but the direction of this bias varies from city to city (Chapelain et al., 2015). In contrast to what has been found for humans, the Colombian spider monkey showed a significant left-side bias for embraces and cheek-to-cheek contacts (Boeving et al., 2017).

A naturalistic observational study of male-female pairs of adults walking along a riverside walkway showed that men were significantly more often on the right from the pair's perspective, i.e., men preferentially kept their female partners on the left side (Rodway and Schepman, 2022). The results of an online survey showed that, unlike women, men report significant side preferences when imagining walking or sitting on a bench with their female partners. The majority of left-handed men imagined keeping their partner on the right side, while the majority of right-handed men imagined their partner on the left-right side (Rodway and Schepman, 2022). No study on non-human primates has specifically focused on positional biases in male-female interactions, but a general tendency to keep social partners on the left rather than the right side has been shown for captive gorillas and chimpanzees (Quaresmini et al., 2014).

### Positional biases in social behavior of ungulates

Lateralization in the positioning of interacting individuals has been studied in a range of domestic and wild ungulates. Saiga antelopes become a particularly interesting model for comparative analysis because, in this species, lateralization in a variety of social interactions was found. Moreover, saigas were studied in the wild excluding the potential confounding effects of domestication, captivity or training (Leliveld, 2019). For that reason, the focus will be primarily on this species.

Observations of spontaneous mother-infant reunions showed that saiga calves prefer to keep the mother to the left when approaching her during traveling and for suckling. Among ungulates, a similar bias was also found in wild reindeer, European bison, muskox, argali sheep, Przewalski's and feral horses (Karenina and Giljov, 2018). The positional bias in saigas' maternal behavior has also been studied, with the majority of mothers preferring to keep their calves on the left side when fleeing (Karenina et al., 2022).

Saiga calves showed a preference to keep other familiar calves (age-mates) on the left rather than the right side (Karenina et al., 2017). A similar bias in positioning relative to a familiar conspecific was also found in adult ungulates, e.g., in domestic horses during affiliative interactions (Farmer et al., 2018) and captive common elands during routine group behaviors (Bordes et al., 2018).

Positional biases in aggressive interactions have been found in saiga male contests. A preference to keep the opponent to the left was found in retreating after fighting and chasing an escaping rival (Giljov et al., 2019). In line with this, left-sided positional preference is evident in stallion fights in Przewalski's and feral horses (Austin and Rogers, 2012, 2014). At the same time, male fallow deer showed a right-sided bias in the termination of lateral displays during contests in the wild (Jennings, 2012). Observations of dominating behaviors within social groups of free-ranging European bison showed that the animals were more likely to display aggressive responses when the herd mate was to the right. The inhibition of aggression, in contrast, occurred more likely when the opponent was to the left (Giljov and Karenina, 2019).

Another type of lateralized social behavior in saiga antelopes is male-female interactions. It has been found that males preferentially keep females on their right side when pursuing them to rejoin with the rest of the group (Giljov et al., 2019).

## Discussion

Lateral biases in the social behavior of non-human primates have demonstrated that human social lateralizations are not unique (Lindell, 2013). However, wider generalizations across mammalian linage, including non-primates, are still rarely found in the literature. Our understanding of human social lateralizations may benefit from more attention to the results on non-primate mammals, especially considering how struggling the interpretation of human studies remains.

The use of forelimbs is lateralized in humans and the extent to which lateral biases in social interactions are determined by motor biases remains unclear. Even after decades of research, the question of the motor vs. emotional nature of cradling bias remains open (Ocklenburg et al., 2018). A large amount of evidence indicates that left-sided cradling bias favors greater right-hemisphere involvement in the monitoring of the infant's state and is generally guided by sensory and emotional rather than motor lateralization (e.g., Manning et al., 1994; Packheiser et al., 2020). At the same time, a stronger right-hand preference is related to a higher likelihood of left-sided cradling (van der Meer and Husby, 2006).

The role of motor biases in other lateralized human interactions is also far from being clear. Several studies tested the link between handedness and head-turning preference during kissing, and the results are conflicting (e.g., Barrett et al., 2006; Ocklenburg and Güntürkün, 2009; Packheiser et al., 2018). In the case of rightward embracing bias, a combination of motor and emotional lateralizations has been suggested (Ocklenburg et al., 2018). A clear effect of handedness has been found in male-female positioning in couples (Rodway and Schepman, 2022). Thus, the involvement of forelimbs in social interactions combined with lateralized forelimb use in humans and other primates makes the interpretation of positional biases in social interactions complicated.

Ungulates are a convenient model for the investigation of social lateralization since they are lacking these confounding factors. When forelimbs do not directly determine the relative positioning of interacting subjects, the interpretation of lateral preferences as manifestations of sensory lateralization becomes much more straightforward. Ungulates became popular models in laterality research partly for casual reasons such as their availability, e.g., as livestock (Leliveld, 2019). However, it is important to emphasize their significance for fundamental studies uncovering the origin of lateral biases in social behavior. The prevalence of lateralized social behaviors demonstrated by the existing evidence together with the variety of social systems and diversity of social behaviors implies that investigations on ungulates may bring important insights into the understanding of social lateralization.

Even a brief comparison of the results of human and ungulate studies suggests some interesting conclusions and directions for future investigation. Left-sided biases in both mother's behavior toward her offspring and the young's positional choices relative to mother and age-mates in saiga antelope and several other ungulate species (Karenina and Giljov, 2018) are congruent with what we see in humans (Harris, 2010; Forrester et al., 2014). The leftward preference in mother-offspring bonding behaviors (Karenina et al., 2017) resembles a left-turn bias specific to parental kissing in humans (Sedgewick and Elias, 2016). A consistency in lateralized behavior of mother and young among mammals suggests ancient evolutionary roots of these invariable biases.

If something in the lateralized social behavior of ungulates can be compared with human embraces, it's allogrooming in horses. In that case, human right-sided bias is not consistent with the results on ungulates (Farmer et al., 2018). and Moreover, human embracing bias is opposite in direction to that (Farmer et al., 2018) in another primate species studied, Colombian spider monkey (Boeving et al., 2017), which do not show a population-level handedness and their forelimb preferences should affect the embracing bias considerably less than they appear to do in humans (Ocklenburg et al., 2018; Packheiser et al., 2020).

In male-female interactions, the direction of lateral biases is not consistent between humans (Rodway and Schepman, 2022) and saigas (Giljov et al., 2019). However, the emotional context of the behaviors studied in the two species is very different (walking pairs vs. male-female pursuit). Observations of stable monogamous pairs in ungulates (other than harem-breeding saigas) are needed for a more appropriate comparison with humans. In the case of aggressive interactions, naturalistic observations of humans are lacking to compare them with the evidence on ungulates.

Social behavior and, in particular, social coordination is assumed to be a driver for the emergence of population-level behavioral biases (Frasnelli and Vallortigara, 2018). Therefore, the intensification of comparative studies may lead to a better understanding of the adaptive function of social lateralization in humans and other species. Social coordination is particularly important within groups and lateralization research on groups of individuals involved in cooperative interactions can provide critically important knowledge for the understanding of fitness benefits associated with lateralization. Future studies on humans and ungulates (as models for comparative analysis) could aim to assess positional biases in multi-individual interactions, e.g., in leader—followers interactions during traveling in groups.

## Author contributions

AG and KK conceptualized and wrote initial draft. All authors contributed to the article and approved the submitted version.
